# The impact of prior outpatient ACE inhibitor use on 30-day mortality for patients hospitalized with community-acquired pneumonia

**DOI:** 10.1186/1471-2466-5-12

**Published:** 2005-09-13

**Authors:** Eric M Mortensen, Marcos I Restrepo, Antonio Anzueto, Jacqueline Pugh

**Affiliations:** 1 VERDICT Research Center, Audie L Murphy VA Hospital, San Antonio, Texas, USA; 2 Division of General Medicine, The University of Texas Health Science Center at San Antonio, USA; 3 Division of Pulmonary and Critical Care Medicine, The University of Texas Health Science Center at San Antonio, USA

## Abstract

**Background:**

Recent studies suggest that angiotensin-converting enzyme (ACE) inhibitors may have beneficial effects for patients at risk for some types of infections. We examined the effect of prior outpatient use of ACE inhibitors on mortality for patients hospitalized with community-acquired pneumonia.

**Methods:**

A retrospective cohort study conducted at two tertiary teaching hospitals. Eligible subjects were admitted with a diagnosis of, had a chest x-ray consistent with, and had a discharge ICD-9 diagnosis of pneumonia. Subjects were excluded if they were "comfort measures only" or transferred from another acute care hospital. Subjects were considered to be on a medication if they were taking it at the time of presentation.

**Results:**

Data was abstracted on 787 subjects at the two hospitals. Mortality was 9.2% at 30-days and 13.6% at 90-days. At presentation 52% of subjects were low risk, 34% were moderate risk, and 14% were high risk. In the multivariable conditional logistic regression analysis, after adjusting for potential confounders, the use of ACE inhibitors at presentation (odds ratio 0.44, 95% confidence interval 0.22–0.89) was significantly associated with 30-day mortality.

**Conclusion:**

Prior outpatient use of an ACE inhibitor was associated with decreased mortality in patients hospitalized with community-acquired pneumonia despite their use being associated with comorbid illnesses likely to contribute to increased mortality. Confirmatory studies are needed, as well as research to determine the mechanism(s) of this protective effect.

## Background

Community-acquired pneumonia is the seventh leading cause of death and the leading cause of infectious death in the United States [1]. Although mortality due to community-acquired pneumonia decreased significantly with the introduction of antibiotics in the 1950s since that time mortality has been stable or increasing [2]. Despite this, only a few new classes of antibiotics have been added to the armamentarium for treating community-acquired pneumonia in the last 20 years and no new classes of medications beyond antibiotics have been added since the 1950s.

Recent studies have demonstrated that elderly patients taking ACE inhibitors have significantly decreased rates of aspiration pneumonia and may have decreased mortality [3-5]. The hypothesized reason for this protective effect is an increase in substance P which accentuates the cough reflex. Other studies have demonstrated significant changes in systemic cytokine levels for subjects treated with ACE inhibitors [6-8]. These cytokines play an important role in host defense mechanisms for patients with community-acquired pneumonia but under certain conditions may lead to septic shock or acute respiratory distress syndrome (ARDS) [9-11].

The study aim was to assess the effects of prior outpatient ACE inhibitor use on 30-day mortality for patients hospitalized with community-acquired pneumonia.

## Methods

This a retrospective cohort study of patients hospitalized with community-acquired pneumonia at 2 academic tertiary care hospitals in San Antonio, Texas. Both hospitals are teaching affiliates of the University of Texas Health Science Center at San Antonio. The Institutional Review Board of the University of Texas Health Science Center at San Antonio approved the research protocol with exempt status.

### Study sites/inclusion and exclusion criteria

We identified all patients admitted to the study hospitals between January 1, 1999 and December 1, 2002 with a primary discharge diagnosis of pneumonia (ICD-9 codes 480.0–483.99 or 485–487.0) or secondary discharge diagnosis of pneumonia with a primary diagnosis of respiratory failure (518.81) or sepsis (038.xx). Subjects were included if they were 1) greater than 18 years of age, 2) had an admission diagnosis of community-acquired pneumonia documented in the medical record, and 3) had a radiographically confirmed infiltrate or other finding consistent with community-acquired pneumonia on chest x-ray or CT obtained within 24 hours of admission.

Exclusion criteria included 1) having been discharged from an acute care facility within 14 days of admission, 2) transfer after being admitted to another acute care hospital, and 3) being comfort measures only on this admission. If a subject was admitted more than once during the study period, only the first hospitalization was abstracted

### Data abstraction

Chart review data included: demographics, comorbid conditions, physical examination findings, laboratory data, and chest radiograph reports. In addition, data on important processes of care measures for patients hospitalized with community-acquired pneumonia were also abstracted: first dose of antibiotics within 8 hours of admission, collection of blood cultures prior to antibiotic administration, and obtaining blood cultures and oxygen saturation measurement within 24 hours of presentation [12]. Antimicrobial therapy was considered guideline-concordant if it agreed with either the 2000 Infectious Diseases Society of America or 2001 American Thoracic Society guidelines [13,14]. Information on all outpatient medications that were either 1) reported as currently being taken by the patient at presentation, or 2) listed in the electronic medical record, were recorded.

Mortality was assessed using information from the Texas Department of Health and Department of Veteran Affairs clinical database. Mortality status was assessed through December 31, 2002.

### Risk adjustment

The pneumonia severity index was used to assess severity of illness at presentation [15]. The pneumonia severity index is a validated prediction rule for 30-day mortality in patients with community-acquired pneumonia. This rule is based on three demographic characteristics, five comorbid illnesses, five physical examination findings, and seven laboratory and radiographic findings from the time of presentation. Patients are classified into five risk classes with 30-day mortality ranging from 0.1% for class I to 27% for class V for patients enrolled in the PORT cohort study [15].

### Outcome

We used 30-day mortality as the outcome for this study. Previous research has demonstrated that 30-day mortality is primarily due to the community-acquired pneumonia rather than other co-existing co-morbid conditions [16,17]. Therefore by using 30-day mortality as our outcome we are able to minimize the effect of the ACE inhibitor use on other co-morbid conditions.

### Statistical analyses

Univariate statistics were used to test the association of sociodemographic and clinical characteristics with all-cause 30-day mortality. Categorical variables were analyzed using the Chi-square test and continuous variables were analyzed using Student's t-test.

A propensity score technique was used to balance covariates associated with ACE inhibitor use between groups [18,19]. The use of the propensity score technique provides a way, in non-randomized studies, to control for pretreatment differences by defining sets of comparable patients. The propensity score was derived from a logistic regression model, and a dichotomous indicator variable indexing whether a patient was on an ACE inhibitor was our dependent variable. The covariates used to develop the propensity score included the pneumonia severity index (which includes comorbid conditions such as congestive heart failure and chronic renal insufficiency), history of hypertension, and history of diabetes mellitus.

A multivariable conditional logistic regression model was derived with 30-day mortality as the dependent variable, and the propensity score as the matching variable [20]. The independent variables in the model were the use of ACE inhibitor at presentation, and process of care measures (initial antibiotics within 8 hours and obtaining blood cultures prior to initial dose of antibiotics, and whether antimicrobial therapy was guideline concordant). Interactions were assessed using cross-product terms between the medications and all of the other variables retained in the models. No significant interactions terms were noted, so they were excluded from the final models. All analyses were performed using STATA version 8 (Stata Corporation, College Station, Texas).

## Results

Data was abstracted on 787 patients at the two hospitals. The mean age was 60 years with a standard deviation of 16 years. The population was 79% male, 84% were admitted through the emergency department, and 20% were admitted to the intensive care unit within the first 24 hours after admission. Mortality was 9.2% at 30-days and 13.6% at 90-days. By pneumonia severity index, 52% were low risk (pneumonia severity index classes I-III), 34% were moderate risk (pneumonia severity index class IV), and 14% were high risk (pneumonia severity index class V). Regarding community-acquired pneumonia-related processes of care, 28% received the initial dose of antibiotics within 4 hours of presentation and an additional 22% received the initial antibiotic dose within 8 hours, 76% of patients had blood cultures obtained within 24 hours and prior to antibiotics, and oxygenation was assessed at presentation in 91%.

Table 1 shows the demographic factors, clinical characteristics, and processes of care data for this population by 30-day mortality. In the univariate analysis numerous individual components of the pneumonia severity index were significantly associated with 30-day mortality including age, nursing home residency, history of congestive heart failure, history of malignancy, altered mental status, systolic blood pressure < 90 mmHg, tachycardia> 125 beats per minute, arterial acidosis, elevated blood urea nitrogen 30 mg/dl, serum sodium < 130 meq/l, and pleural effusion on chest radiograph. The only processes of care that were statistically significant were the assessment of oxygenation within 24 hours or presentation and use of guideline-concordant antibiotics. ACE inhibitor use was significantly associated (p = 0.05) with 30-day mortality.

**Table 1:** Subject Demographic and Clinical Characteristics by 30-Day Mortality*

	**30-Day Mortality**		
**Variable**	**Alive (n = 714)**	**Dead (n = 72)**	**p-value**
Age, years +/- standard deviation	60.2+/-16.4	62.9 +/-16.4	0.09
Men	561 (79)	60 (83)	0.3
Nursing home resident	41 (6)	13 (18)	<0.001
Emergency department admission	598 (84)	58 (81)	0.5
Admitted to intensive care ≤ 24 hours	118 (17)	36 (50)	<0.001
***Preexisting Comorbid Conditions***			
Congestive heart failure	105 (15)	18 (25)	0.02
Chronic pulmonary disease	195 (27)	23 (31)	0.4
History of stroke	93 (13)	12 (17)	0.4
Chronic liver disease	83 (12)	11 (15)	0.4
History of malignancy	58 (8)	20 (28)	<0.001
Renal insufficiency	74 (10)	13 (18)	0.05
***History, Physical, Laboratory, and Radiographic Data***			
Altered mental status	68(10)	17(24)	<0.001
Respiratory rate > 30 per minute	71 (10)	11 (15)	0.2
Systolic blood pressure < 90 mmHg	16 (2)	5 (7)	0.02
Heart rate > 125 per minute	86 (12)	19 (26)	0.001
Temperature < 95° or > 104°	19 (3)	2 (3)	0.9
Arterial pH < 7.35	37 (5)	12 (17)	<0.001
Arterial oxygenation < 90%	149 (21)	27 (38)	0.001
Hematocrit < 30%	64 (9)	8 (11)	0.5
Blood urea nitrogen > 30 mg/dL	135 (19)	33 (46)	<0.001
Serum glucose > 250 mg/dL	71 (10)	5 (7)	0.4
Serum sodium < 130 meq/L	98 (14)	18 (25)	0.01
Pleural effusion	160 (11)	29 (35)	0.001
***Pneumonia Severity Index***			
Class I-III	393 (54)	16 (22)	
Class IV	240 (34)	26 (36)	
Class V	82 (12)	30 (42)	<0.001
***Processes of Care***			
Initial antibiotics within 4 hours	201 (28)	22 (31)	0.7
Initial antibiotics within 8 hours	358 (50)	36 (50)	1.0
Blood cultures prior to antibiotics	540 (76)	55 (76)	0.9
Oxygenation assessed ≤ 24 hours	538 (75)	65 (90)	0.004
Guideline-concordant antibiotics	574 (80)	51 (71)	0.05
***Outpatient Medications***			
ACE inhibitor	183 (25)	11 (15)	0.05

* Data are presented as number (%) or mean +/-standard deviation

Table 2 demonstrates clinical and demographic variables and the association with use/non-use of ACE inhibitors at presentation. Co-morbid conditions and demographic variables significantly associated with ACE inhibitor use included coronary artery disease, higher age, diabetes mellitus, chronic obstructive pulmonary disease, not currently smoking, congestive heart failure, prior stroke, no history of liver disease, and chronic renal disease. Physical exam and laboratory findings associated with ACE inhibitor use included decreased numbers of patients with heart rates > 125, higher rates of elevated blood urea nitrogen > 30 mg/dL, lower rates of serum hematocrit < 30%, and higher rates of serum glucose > 250 mg/dL. Of the 787 subjects 194 (25%) were on ACE inhibitors. There was greater ACE inhibitor use in those who were in the moderate (30%, n = 74) and severe (34%, n = 38) risk groups versus those with low severity of illness (19%, n = 77), p < 0.001.

**Table 2:** Use versus non-use of ACE inhibitors by demographic and clinical characteristics*

	**ACE Inhibitor Use**		
**Variable**	**Not on ACE inhibitor** ** (n= 593)**	**On ACE inhibitor ** **(n=194)**	**p-value**
Age, years +/- standard deviation	58.2 +/-16.9	67.1+/-13.6	<0.001
Men	467 (79)	154(80)	0.8
Nursing home resident	43(7)	11(6)	0.4
Emergency department admission	498(84)	158(81)	0.38
Admitted to intensive care ≤ 24 hours	116(20)	38(20)	1.0
***Preexisting Comorbid Conditions***			
Diabetes Mellitus	138(23)	92(47)	<0.001
Coronary artery disease	132(22)	98(51)	<0.001
Chronic pulmonary disease	151(25)	67(35)	<0.001
Current tobacco use	200(31)	35(18)	<0.001
Congestive heart failure	59(10)	64(33)	<0.001
History of stroke	67(11)	38(20)	0.003
Chronic liver disease	82(14)	12(6)	0.004
History of malignancy	57(10)	21(11)	0.6
Renal insufficiency	54(9)	33(17)	0.002
***History, Physical, Laboratory, and Radiographic Data***			
Altered mental status	62(10)	23(12)	0.6
Respiratory rate > 30 per minute	67(11)	15(8)	0.16
Systolic blood pressure < 90 mmHg	17(3)	4(2)	0.5
Heart rate > 125 per minute	87(15)	18(9)	0.06
Temperature < 95° or > 104°	18(3)	3(2)	0.26
Arterial pH < 7.35	33(6)	16(8)	0.18
Arterial oxygenation < 90%	128(22)	48(25)	0.4
Hematocrit < 30%	59(10)	13(7)	0.17
Blood urea nitrogen > 30 mg/dL	110(19)	58(30)	<0.001
Serum glucose > 250 mg/dL	50(8)	26(13)	0.04
Serum sodium < 130 meq/L	95(16)	21(11)	0.08
Pleural effusion	141(24)	48(25)	0.8
***Pneumonia Severity Index***			
Class I-III	332(56)	77(40)	
Class IV	187(32)	79(41)	
Class V	74(12)	38(20)	<0.001

* Data are presented as number (%) or mean +/-standard deviation

In the multivariable conditional logistic regression analysis after adjusting for potential confounders the use of ACE inhibitors at presentation (odds ratio (OR) 0.44, 95% confidence interval (CI) 0.22–0.89) was associated with 30-day mortality. The other variables in the model included initial antibiotics within 4 hours (OR 1.09, 95% CI 0.64-1.85), obtaining blood cultures prior to antibiotics (OR 1.10, 95% CI 0.6-1.9), oxygenation assessment within 24 hours (OR 1.31, 95% CI 0.5–3.4), and use of guideline-concordant antimicrobial therapy (OR 0.6, 0.35–1.05).

## Discussion

We found that prior outpatient use of ACE inhibitors was associated with decreased 30-day mortality for subjects hospitalized with community-acquired pneumonia. Our findings provide further support to other studies that demonstrate that ACE inhibitor use is associated with decreased mortality for patients with pneumonia.

There are several potential mechanisms for a beneficial effect on mortality for patients on ACE inhibitors. ACE inhibitors have been shown in clinical studies to increased serum levels of substance P, which is hypothesized to lead to a better gag reflex and increased clearance of secretions [3-5]. Our study specifically excluded patients with a discharge diagnosis of aspiration pneumonia; however, this diagnosis can be clinically difficult to distinguish from community-acquired pneumonia. Other studies have demonstrated that ACE inhibitors have significant immunomodulatory effects on circulating cytokines [6-8]. Therefore the beneficial effect may be due to blunting of the cytokine response so that these patients have lower rates of sepsis and/or ARDS.

Although our study was retrospective and subject to the recognized limitations of such studies, we carefully assembled our cohort from complete patient discharge data to avoid ascertainment bias. Additionally, during chart abstraction we encountered a very small amount (<5%) of missing data. Our sample was predominantly men due to the inclusion of a VA hospital and it is possible, but unlikely, that women may have differential responsiveness to ACE inhibitors as compared to men. In addition we are unable to examine whether or not there was significant amounts of non-prescription or non-compliance of ACE inhibitor therapy due to economic considerations. Also we are unable to assess factors such as inpatient continuation of the ACE inhibitor or the dose effect due to the design of this study. Further research is needed to examine these factors. Finally, as in any non-experimental study, we are unable to state conclusively that the prior outpatient use of ACE inhibitors is the cause of decreased mortality in this cohort. However, since patients on ACE inhibitors had significantly higher severity of illness scores at presentation we feel that we have good evidence that these medications may have beneficial effects for patients hospitalized with community-acquired pneumonia.

## Conclusion

In conclusion, our study finds that prior outpatient use of ACE inhibitors reduces mortality for patients with community-acquired pneumonia. Our results add further strength to the existent recommendations to use ACE inhibitors in patients with congestive heart failure, diabetes mellitus, and renal disease, since these patients are at higher risk for either contracting pneumonia or dying from pneumonia when they do contract it. Future research, especially randomized clinical trials, are needed to examine whether either ACE inhibitors are protective when used in an inpatient setting for patients lacking traditional indications for the use of these medications.

## Competing interests

The author(s) declare that they have no competing interests.

## Authors' contributions

EMM originated and coordinated the study, obtained funding, contributed to the analysis of the data, and preparation of the paper.

MIR contributed to the design of the study, contributed to the analysis of the data, and preparation of the paper.

AA contributed to the design of the study, contributed to the analysis of the data, and preparation of the paper.

JP contributed to the design of the study, contributed to the analysis of the data, and preparation of the paper.

## Pre-publication history

The pre-publication history for this paper can be accessed here:


